# ﻿*Solanum stellaticalycinum*, a new simple-leaved species of the Pteroidea clade from Peru (Potato Clade, *Solanum*, Solanaceae)

**DOI:** 10.3897/phytokeys.266.166870

**Published:** 2025-11-10

**Authors:** Marco A. Cueva Manchego, Italo F. Treviño, Diego A. Sotomayor, María I. Villalba, Sandra Knapp

**Affiliations:** 1 Laboratorio de Florística, Departamento de Dicotiledóneas, Museo de Historia Natural, Universidad Nacional Mayor de San Marcos (UNMSM), Av. Arenales 1256, Lima 11, Peru Universidad Nacional Mayor de San Marcos (UNMSM) Lima Peru; 2 Universidad Tecnológica del Peru (UTP), Tacna y Arica 160, 04001, Arequipa, Peru Universidad Tecnológica del Peru (UTP) Arequipa Peru; 3 Departamento de Ingeniería Ambiental, Facultad de Ciencias, Universidad Nacional Agraria La Molina (UNALM), Av. La Molina s/n, Lima, Peru Universidad Nacional Agraria La Molina (UNALM) Lima Peru; 4 Servicio Nacional de Áreas Naturales Protegidas (SERNANP), Los Petirrojos 355, San Isidro 15036, Lima, Peru Servicio Nacional de Áreas Naturales Protegidas (SERNANP) Lima Peru; 5 Natural History Museum, Cromwell Road, London SW7 5BD, UK Natural History Museum London United Kingdom

**Keywords:** Andes, cloud forest, *
Solanum
*, taxonomy, Yanachaga-Chemillén

## Abstract

A new species from the montane forests in central Peru belonging to the Pteroidea clade of *Solanum* is described and illustrated. *Solanum stellaticalycinum* M.A.Cueva, Treviño & D.Sotomayor, **sp. nov.** is known only from the Yanachaga-Chemillén National Park and adjacent areas (Prov. Oxapampa, Dept. Pasco, Peru). With simple leaves, it is morphologically similar to *S. anceps* Ruiz & Pav., a widespread species in South America, and to *S. angustialatum* Bitter and *S. incurvum* Ruiz & Pav., both known from the montane forests of northern and central Peru. *Solanum stellaticalycinum* is distinguished from all these by its purple pedicels and flowers with linear to narrowly triangular calyx lobes, a membranous corolla with lanceolate lobes and ovoid-conical, smooth or slightly rugose fruits. Here we discuss the taxonomic affinities, distribution and conservation status of this new species. We also include a key to all species of Pteroidea with complete distributions at the country level and department level distributions within Peru.

## Abstract (es)

Se describe e ilustra una nueva especie de los bosques montanos del centro del Peru, perteneciente al clado Pteroidea de *Solanum*. *Solanum stellaticalycinum* M.A.Cueva, Treviño & D.Sotomayor, **sp. nov.** se conoce únicamente del Parque Nacional Yanachaga-Chemillén y áreas adyacentes (Prov. Oxapampa, Depto. Pasco, Perú). Con hojas simples, es morfológicamente similar a *S. anceps* Ruiz & Pav., una especie ampliamente distribuida en Sudamérica, y a S. angustialatum Bitter y *S. incurvum* Ruiz & Pav., ambas conocidas de los bosques montanos del norte y centro del Perú. *Solanum stellaticalycinum* se distingue de todas estas por sus pedicelos morados y flores con lóbulos del cáliz lineales a estrechamente triangulares, una corola membranosa con lóbulos lanceolados y frutos ovoide-cónicos, lisos o ligeramente rugosos. Aquí discutimos las afinidades taxonómicas, la distribución y el estado de conservación de esta nueva especie. También incluimos una clave para todas las especies de Pteroidea con distribuciones completas a nivel de país y distribuciones a nivel de departamento dentro del Perú.

## ﻿Introduction

*Solanum* L. (Solanaceae) is a cosmopolitan genus and the most species-rich within the family Solanaceae. It comprises approximately 1,250 species worldwide, and it is considered one of the most species-rich genera of flowering plants (Moonlight et al. 2024). Many species of *Solanum* are cultivated and of economic importance as sources of food (e.g., potatoes, tomatoes) and alkaloids for the pharmaceutical industry (Hawkes 1999). Species of *Solanum* are found in a variety of habitats, but the greatest species richness is concentrated in the tropics of South America (Knapp 2002; Hilgenhof et al. 2023). *Solanum* species are herbs, shrubs, and even large trees, with pentamerous flowers, fused sepals and petals, star-shaped to rotate corollas, stamens with short filaments and connivent anthers with poricidal dehiscence (Nee 1999; Bohs 2005; Hilgenhof et al. 2023). In light of its large number of species and varied morphology, *Solanum* has been classified under different systems at the infrageneric level (D’Arcy 1972; Child 1990, 1998; Nee 1999) within which several sections or groups of species have been identified based on morphological studies. Recently, studies using molecular markers have confirmed the monophyly of many of these, and a system of informal clade names has been established (Gagnon et al. 2022; Hilgenhof et al. 2023).

The Pteroidea clade (Solanum section Pteroidea Dunal) is a monophyletic group (Tepe et al. 2016; Gagnon et al. 2022) comprising ten erect herbaceous or climbing species, with simple or compound (deeply pinnatifid without interstitial leaflets) leaves and with axillary scorpioid inflorescences, many of the species have rugose conical fruits (Knapp and Helgason 1997). The axillary inflorescences found in all species of the group are unique in *Solanum* and are the distinguishing apomorphy of the clade (Hilgenhof et al. 2023). Species of the group are found from Mexico to Bolivia, with a centre of diversity in the eastern Amazonian premontane or montane forests in Peru and Ecuador (Knapp and Helgason 1997). Recent DNA studies support the monophyly of group and its relationship with Solanum section Herpystichum Bitter, both considered within the larger clade informally known as the “Potato clade”, which also includes the wild relatives of the potato, tomato and pepino (Bohs 2005; Tepe and Bohs 2010; Tepe et al. 2016).

In the most recent taxonomic review of the group (Knapp and Helgason 1997), the ten known species were divided into two informal groups: 1) the “*Solanum ternatum* group”, with *S. incurvum* Ruiz & Pav. and *S. ternatum* Ruiz & Pav., characterized by larger fleshy corollas up to 2 cm in diameter at anthesis with triangular lobes and globose and smooth fruits with numerous flat and reniform seeds; and 2) the “*Solanum mite* group”, with *S. anceps* Ruiz & Pav., *S. angustialatum* Bitter, *S. chamaepolybotryon* Bitter, *S. conicum* Ruiz & Pav., *S. mite* Ruiz & Pav., *S. savanillense* Bitter, *S. trizygum* Bitter, and *S. uleanum* Bitter, all characterized by having small membranous corollas less than 1 cm in diameter at anthesis with strongly very reflexed lobes, rugose conical fruits (except for *S. mite* that possesses globose fruits), and with few ovoid reniform seeds. Of these species only *S. anceps*, *S. angustialatum*, and *S. incurvum* have simple leaves, the rest all have imparipinnate pinnatifid leaves with three to more leaflets (Knapp and Helgason 1997). *Solanum anceps* is one of the species with the widest distribution inhabiting the Amazonian region and montane forests of Peru and Bolivia up to 2,000 m in elevation. *Solanum angustialatum* is only known from premontane forests in the Department of San Martín in Peru. *Solanum incurvum* grows in cloud forests close to 3,000 m in elevation and is poorly collected; it is known just from a few localities in southern Ecuador and central Peru (Knapp and Helgason 1997).

While undertaking taxonomic revision of the Solanaceae in the Yanachaga-Chemillén National Park, we collected several specimens of an unusual species of *Solanum* that we recognized as a simple-leaved member of the Pteroidea clade distinctly different from any currently known species in the group. Here we describe this new species and discuss its distribution, relationships, and conservation status.

## ﻿Materials and methods

This study was conducted primarily using field-collected material. We analyzed living plants of all species of the Pteroidea clade with simple leaves and herbarium specimens available at the following herbaria: AMAZ, BM, CUZ, HAO, HOXA, HUT, HUSA, K, MA, MOL and USM. We also examined the types of *S. anceps* and *S. incurvum* at MA, and *S. angustialatum* at K. Fine-scale morphological details were observed with stereoscopic and compound microscopy. Seeds were extracted from mature fruits, air-dried and mounted on aluminum stubs previously furnished with carbon adhesive tape on both sides. Mounted seeds were then covered with gold-palladium in a Denton Vacuum Dex II metal-supplier for approximately 60 seconds and then observed with a Phillips XL 20 Scanning Electron Microscope (SEM) at the Center for Electron Microscopy of Universidad Nacional de San Agustín, Arequipa, Peru. Distribution of *S. stellaticalycinum* is based on geographical information from herbarium specimens; where coordinates were not recorded on labels they were calculated using Google Earth. A preliminary conservation status was assessed using the IUCN (2022) criteria. All specimens were collected in accordance with Peruvian regulations under permits 004-2013-SERNANP-DGANP-JEF and 026C/C-2008-INRENA-IANP issued by the national environmental authority (SERNANP).

## ﻿Taxonomic treatment

### ﻿ Solanum stellaticalycinum

Taxon classificationPlantaeSolanalesSolanaceae

M.A.Cueva, Treviño & D.Sotomayor sp. nov.

6BC1FD64-C1CC-59CC-BD84-6160ECC54ED5

urn:lsid:ipni.org:names:77371677-1

Figs 1, 2, 3

#### Diagnosis.

*Solanum stellaticalycinum* differs from all other species of the Pteroidea clade in its calyx with narrow linear lobes and corolla with narrow lanceolate lobes with elongate, acuminate apices that are spreading at anthesis. It differs from the sympatric *S. anceps* Ruiz & Pav. in its larger flowers and fruits on pendent rather than erect pedicels, and from *S. mite* Ruiz & Pav. and *S. conicum* Ruiz & Pav. in its simple, rather than deeply pinnatifid, leaves.

#### Type.

Peru • Pasco: Prov. Oxapampa, Distrito de Huancabamba, Parque Nacional Yanachaga‐Chemillén, Sector Abra Yanachaga, camino hacia parcela, 10°22'48"S, 75°27'45.25"W, 2,880 m, 2 Oct 2009, *M. Cueva*, *A. Monteagudo*, *A. Peña*, *J. Mateo*, *R. Rivera* & *V. Peña 645* (holotype: HUSA [acc. # 10917]; isotypes: USM [acc. # 253156], HOXA, MO [MO-4348867, acc. # 7105417], HUT).

#### Description.

Erect, single-stemmed herbs or subshrubs, 0.4–2 m tall. Stems 0.4–1.3 cm in diameter, terete; young growth minutely papillose glandular; internodes 0.7–1.2 cm long, brownish green, pubescent with whitish gray 7-8-celled simple uniseriate trichomes 0.5–0.7 mm long; older internodes 1.3–2.7 cm long, glabrous or glabrescent, dark brownish green. Sympodial units unifoliate. Leaves simple, entire; blades 7–10 cm long, 1.6–4.3 cm wide, elliptic to ovate, membranous, discolorous, both surfaces glabrous or with a few simple uniseriate trichomes ca. 0.5 mm long along the veins and minutely papillate with tiny glandular hairs; adaxial surface dark green and shiny (dark brown when dry); abaxial surface pale green or with purple veins (yellowish brown when dry); principal veins 9–10 pairs, slightly raised on the abaxial surface; base cuneate to attenuate; margins entire; apex acute to acuminate; petioles 0.4–1.2 cm long, green or purple, glabrous, with tiny papillae like those of the stems and leaves. Inflorescences axillary scorpioid cymes, (7–) 10–25 mm long, with 4–13 flowers generally inserted in the distal 1/3 of the axis,1–4 flowers open at a time; peduncle 6–17 mm long, dark purple, shiny, glabrous or glabrescent, with papillate trichomes like those of stems and leaves; pedicels 10–14 (–16) mm long at anthesis, ca. 0.8 mm in diameter at the base widening to ca. 2 mm in diameter at the apex, dark purple, glabrous or with some dispersed simple uniseriate trichomes like those of the stems and leaves, articulated at the base, deflexed at maturity so the flowers face downwards; pedicel scars slightly raised, closely spaced. Buds tapering-ellipsoid, enclosed within the linear calyx lobes. Flowers 5-merous, cosexual. Calyx with the tube obconical, ca. 2 mm long, 3–3.4 mm in diameter, dark purple, shiny, glabrous or sparsely white-pubescent with 6-7-celled simple uniseriate trichomes up to 0.4 mm long and some tiny papillae, the lobes (2–) 3.5–4.5 mm long and 0.5 mm wide, linear to narrowly triangular, dark purple with white or green margins, glabrescent with whitish 6-7-celled simple uniseriate hairs abaxially, brown-glandular papillae on the proximal half and extending towards the distal portion of the calyx tube adaxially. Corolla 17–20 mm in diameter at anthesis, deeply stellate, divided almost to the base, the lobes 8–11.5 mm long, ca. 2 mm wide, lanceolate, spreading at anthesis, membranous, white with dark purple spots, the margins puberulent with 1-3-celled simple uniseriate hairs, less than 0.2 mm long, both surfaces glabrous, the apex elongate-acuminate. Stamens equal; filament tube minute; free portion of the filaments ca. 1.5 mm long, glabrous, white; anthers 3–4 mm long, 1.5–2 mm wide, oblong to oblong-ellipsoid, yellow, poricidal at the tips, the pores introrse, lengthening to slits with age. Ovary conical ca. 5 mm long and 1 mm in diameter, glabrous; style 6–6.5 mm long, ca. 0.5 mm in diameter, exserted 2.5–3.5 mm beyond the anthers, white, straight, densely pubescent in the basal half with 1-3-celled simple uniseriate hairs up to 0.2 mm long; stigma ca. 0.5 mm long, capitate and slightly bilobed, white, the surface minutely papillose. Fruit an ovoid-conical berry, 1.3–2.1 (–2.7) cm long, 0.9–1.3 (–1.7) cm in diameter, yellowish green, the pericarp shiny, smooth or slightly rugose; fruiting pedicels 1.7–1.8 cm long, deflexed to pendent, the calyx persistent, the lobes 3–4 mm long, linear to narrowly triangular. Seeds 18–27 per berry, ovoid-reniform, ca. 3 mm long, 2.5 mm wide, brownish green, the testal cells deeply sinuate, with anticlinal walls ca. 10 µm in thickness; stone cells absent. Chromosome number: not known.

#### Distribution

**(Fig. 4).***Solanum stellaticalycinum* is endemic to the Department of Pasco (Prov. Oxapampa) in Peru. All specimens have been collected in the Yanachaga-Chemillén National Park and surrounding areas (Fig. 4), in the sectors of Abra Yanachaga, San Alberto and Chacos.

#### Ecology and habitat.

**Figure 1.**
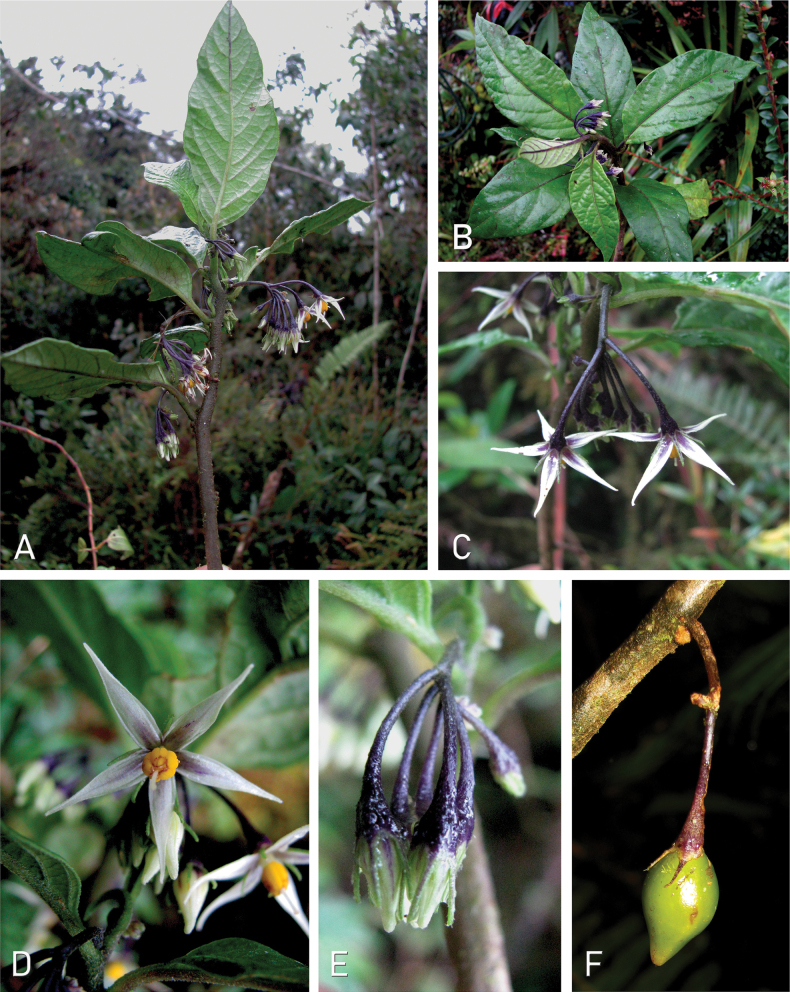
*Solanum stellaticalycinum* M.A.Cueva, Treviño & D.Sotomayor. A, B. Habit; C. Inflorescence; D. Flower at full anthesis; E. Floral buds; F. Fruit (*M. Cueva et al. 645*). Photos by M.A. Cueva.

**Figure 2.**
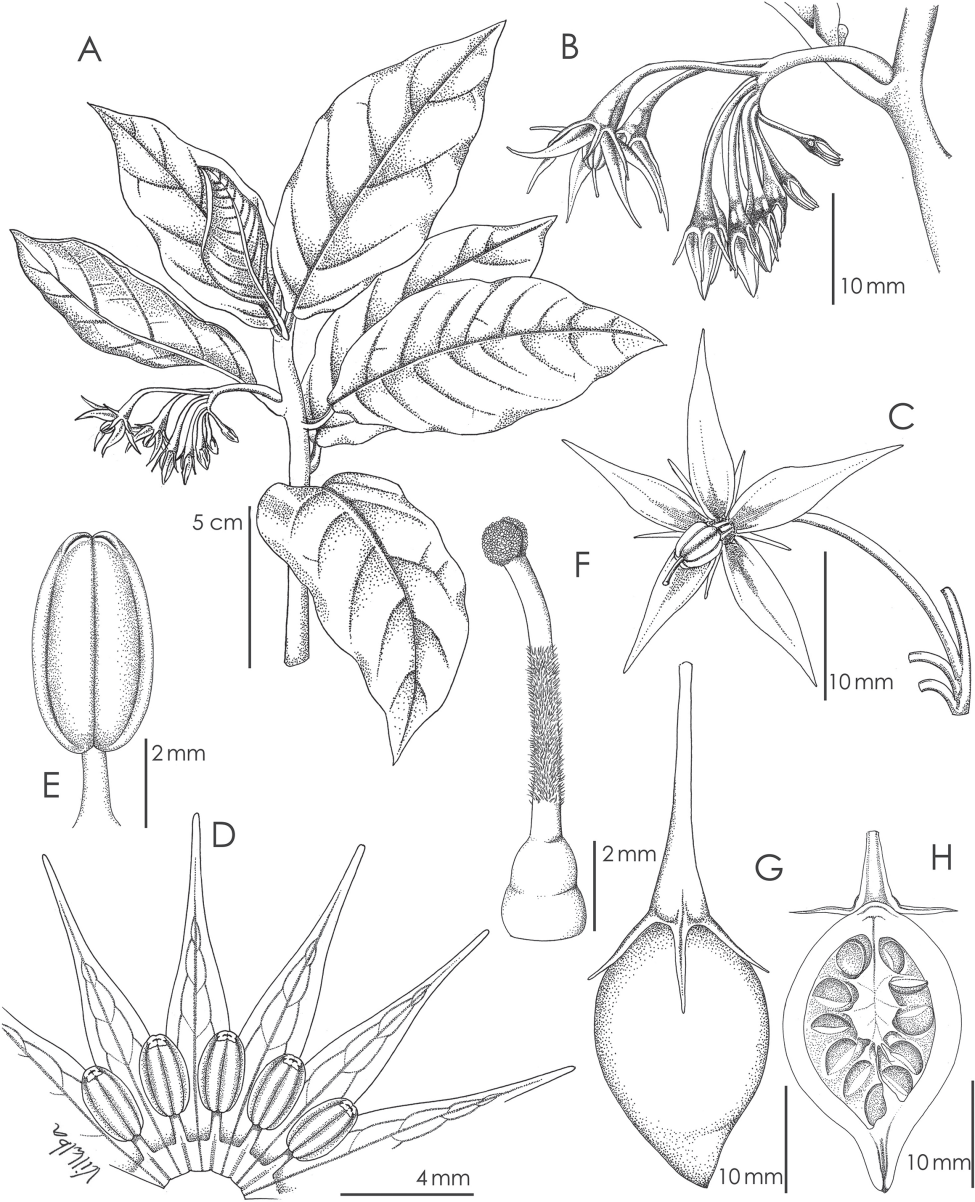
*Solanum stellaticalycinum*. A. Habit; B. Inflorescence; C. Flower; D. Floral dissection; E. Anther detail; F. Gynoecium detail. G, H. Fruit longitudinally dissected to reveal seeds (based on *M. Cueva et al. 645*). Illustration by Maria I. Villalba.

*Solanum stellaticalycinum* grows as a single stemmed herb (Figs 1A, 2A) in the understory of primary montane forests, often close to the somewhat open borders of rural paths, between 2,399 and 3,000 m elevation. Plants have been collected in flower between August and November (Fig. 1A), and fruiting specimens in April and October. It is likely that these plants bloom irregularly all year round, as do many other solanums from similar habitats.

**Figure 3.**
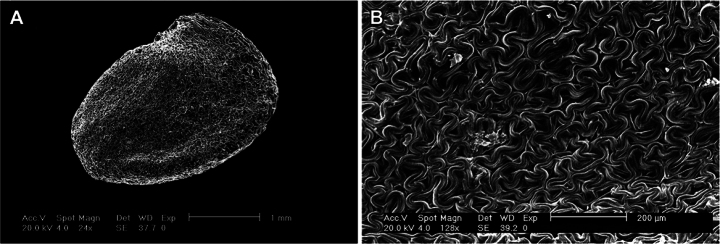
Images of the seed coat using SEM from *Solanum stellaticalycinum*. A. Whole seed; B. Testal cell shape detail. Scale bar in image.

**Figure 4.**
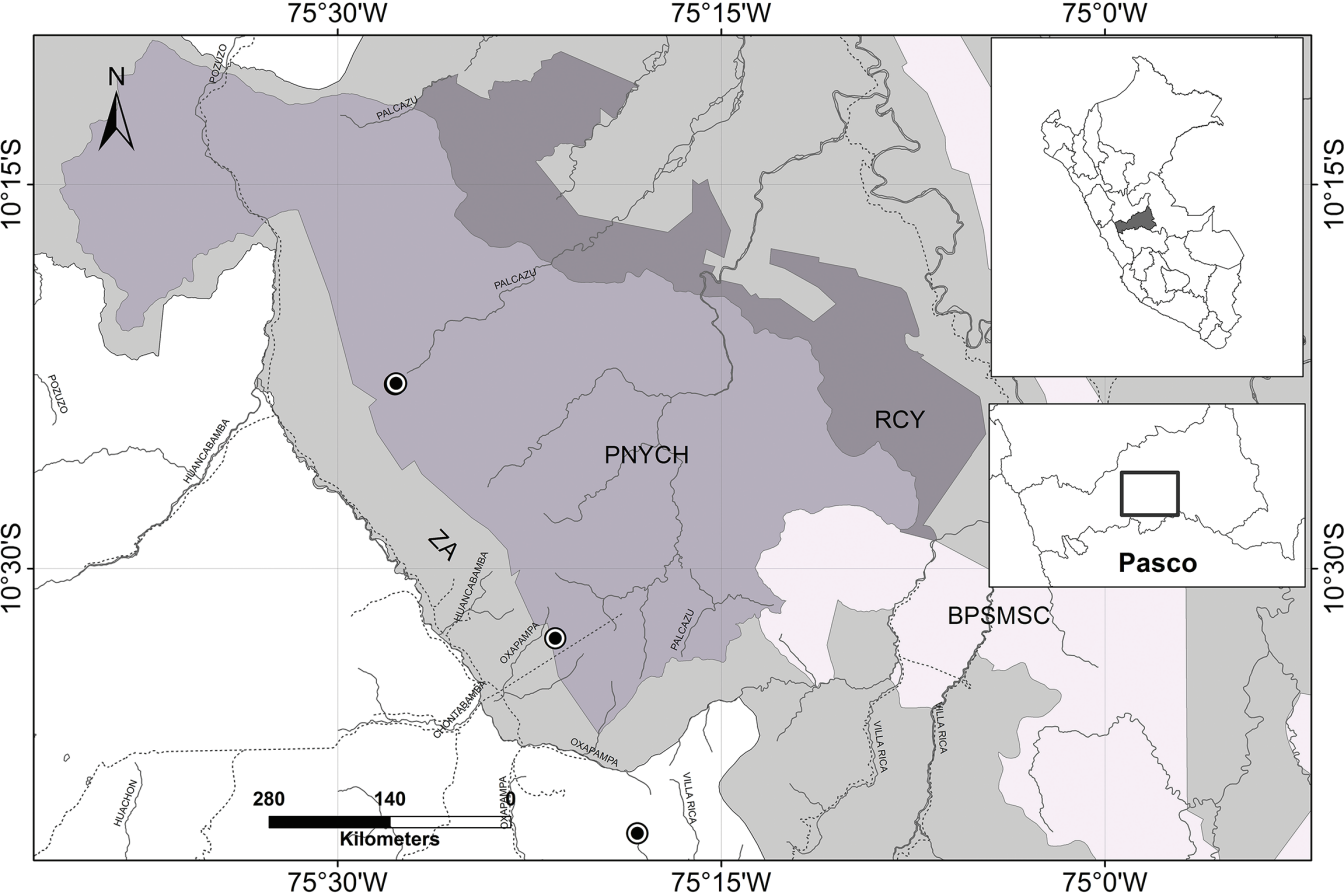
Distribution of *Solanum stellaticalycinum*. Abbreviations: Yanachaga‐Chemillén National Park (PNYCH), Buffer Zone (ZA), Reserva Comunal Yanesha (RCY), Bosque de Protección San Matías San Carlos (BPSMSC).

#### Etymology.

The species epithet refers to the shape of the calyx, which is like a star (see Figs 1, 2). This character is not present in any other species of the Pteroidea clade.

#### Preliminary conservation status

**(IUCN 2022).***Solanum stellaticalycinum* is here assigned a preliminary threat status of Endangered (EN), using the criteria B1ab (i, iii). It is only known from three localities, and the known range (EOO) is approximately 111.9 km^2^. It is likely that this species is distributed along the entire extension of the Yanachaga range, which occurs within the Yanachaga-Chemillén National Park, an area protected by the Peruvian government. It possible however, that in the future, the area of occupancy of this species will shrink in response to habitat modification resulting from human activities in buffer zone areas of the park.

#### Additional specimens examined

**(*paratypes*). Peru** • Pasco: Prov. Oxapampa: Dist. Oxapampa, Yanachaga-Chemillén National Park, San Alberto sector, surroundings of Refugio el Cedro, 2483 m, 10°32'43.3"S, 75°21'29.5"W, 26–27 Apr 2009, *M. Cueva* & *R. Rivera 614* (USM); New highway between Oxapampa-Bosque Shollet-Villa Rica, 2399 m, 25 Oct 2013, *M. Cueva* & *S. Smith 1700* (HUSA); • Dist. Huancabamba, Abra Yanachaga, Yanachaga-Chemillén National Park, 2900–3000 m, 10°22'46"S, 75°27'43"W, 23 Nov 2004, *A. Monteagudo*, *A. Peña*, *J. Perea*, *R. Francis*, *J. L. Mateo & E. Becerra 7840* (BM [BM015735503], HOXA [acc. # 00012525], USM, HUT, AMAZ, MOL, MO [MO-2265895, acc. # 6217980]); • Dist. Huancabamba, Abra Yanachaga, Yanachaga-Chemillén National Park, 2930 m, 10°22'46.3"S, 75°27'42.9"W, 03 Aug 2005, *E. Ortiz* & *R. Francis735* (HOXA [acc.# 00018108], USM, MO [MO-1662368, acc. # 4824338]).

#### Discussion.

*Solanum stellaticalycinum* is not easily confused with any other species of Pteroidea. The flowers with linear calyx lobes alternating with the lanceolate corolla lobes (Fig. 1D) are unique in the clade, and unusual in *Solanum* overall. It shares membranous corollas, conical ovoid fruits with smooth or rugose surfaces, and ovoid reniform seeds with irregularly shaped testal cells with sinuate anticlinal walls (Fig. 3) with other members of the *S. mite* species group (sensu Knapp and Helgason 1997), where only *S. anceps* and *S. angustialatum* have simple leaves. The other six known taxa in the *S. mite* group (*S. conicum*, *S. chamaepolybotryon*, *S. mite*, *S. savanillense*, *S. trizygum* and *S. uleanum*) have compound imparipinnatifid leaves with three or more leaflets (Knapp and Helgason 1997). *Solanum anceps* and *S. angustialatum* are morphologically similar and both differ from *S. stellaticalycinum* in their whitish green pedicels 4–7 mm long (versus purple pedicels 10–14 mm long, Figs 1E, 2B, C), very small flowers with delate calyx lobes up to 1 mm long (versus linear calyx lobes 3.5–4.5 mm long, Figs 1C, F, 2), corollas at anthesis 3–10 mm in diameter with very reflexed membranous triangular lobes 3–5 mm long (versus corolla at anthesis 17–20 mm in diameter, with extended, membranous, narrowly lanceolate lobes 8–11.5 mm long, Figs 1C–F, 2B, C ,G and anthers ca. 2 mm long (versus 3–4 mm long in *S. stellaticalycinum*, Fig. 2E).

*Solanum stellaticalycinum* is also somewhat similar to the sympatric *S. incurvum* from the *S. ternatum* group (sensu Knapp and Helgason 1997). *Solanum incurvum* differs from *S. stellaticalycinum* in its calyx with deltate lobes ca. 1 mm long (versus linear lobes 3–4 mm long), a larger fleshy corolla generally 23–25 mm in diameter with triangular lobes (versus membranous corollas 10–14 mm in diameter), and in its globose smooth fruits with flattened reniform seeds (versus conical fruits with ovoid reniform seeds in *S. stellaticalycinum*). Some of the examined individuals of *S. anceps* and *S. incurvum* exhibit variation regarding fruit texture and corolla consistency. Some specimens of *S. anceps* develop smooth ovoid fruits with only slight whitish rugosities, similar to those seen in some specimens of *S. stellaticalycinum*, but the pedicels are still short and erect rather than pendent, and corolla diameter does not exceed 10 mm in diameter. In some individuals of *S. incurvum*, the corollas can develop a membranous consistency, and fruits can be ovoid (and somewhat flattened) in the first stages of their development, but the deltate or triangular shape of both calyx and corolla lobes, as well as the flattened reniform seeds are distinctive. Morphological differences amongst the simple-leaved species of Pteroidea are summarized in Table 1.

**Table 1.** Morphological differences between the simple-leaved members of Pteroidea: *Solanum stellaticalycinum*, *S. anceps*, *S. angustialatum*, and *S. incurvum*.

Attributes	S. stellaticalycinum	S. anceps	S. angustialatum	S. incurvum
Habit	Single‐stemmed shrub or herb	Single‐stemmed shrub or herb	Single‐stemmed shrub or herb	Climbing or single‐stemmed shrub or herb
Stem form	Terete	Terete	Angular or winged	Terete
Mature leaf width	1.6–4.3 cm	5–18.5 cm	4–15 cm	3.9–10.5 cm
Petiole form	Cylindrical canaliculate	Cylindrical canaliculate	Winged	Cylindrical canaliculate
Petiole length	0.4–1.2 cm	1–5 cm	0–3 cm	4–12.5 cm
Bud shape	Conical	Ovoide or globose	Globose	Ovoid, ellipsoid
Corolla in bud	Enclosed in calyx lobes	Exserted from calyx lobes	Exserted from calyx lobes	Exserted from calyx lobes
Corolla diameter	17–20 mm	5–7 mm	4–5 mm	12–25 mm
Corolla texture	Membranous	Membranous	Membranous	Fleshy
Corolla lobes at anthesis	Spreading	Strongly reflexed	Strongly reflexed	Spreading
Calyx lobe shape	Linear to narrowly triangular	Deltate, tips acute	Deltate, tips rounded	Deltate, tips acute to obtuse
Calyx lobe length	3.5–4.5 mm	0.5–1 mm	0.5 mm	1–2 mm
Fruit position	Deflexed to pendent	Erect	Erect	Pendent
Fruit shape	Ovoid-conical	Conical	Conical	Globose
Fruit surface	Smooth or slightly rugose	Rugose	Rugose	Smooth
Seed shape	Ovoid‐reniform	Ovoid‐reniform	Ovoid‐reniform	Flattened‐reniform
Seed number per berry	18–27	ca. 40	ca. 20	ca. 80–100
Testal cell walls	Sinuate	Sinuate	Sinuate	Straight (rectangular)

### ﻿Artificial key to all species of the Pteroidea clade

[Note: Total country distribution noted for each species noted; distribution to Department level included for Peru. An online multiaccess key for all species of *Solanum*, including Pteroidea, can also be found at https://solanum-species.identificationkey.org/mkey.html.]

**Table:** 

1	Leaves simple (undivided) with entire margins	**2**
–	Leaves pinnatifid (apparently pinnate or ternate), with distinct leaflets	**5**
2	Corollas 17–25 mm in diameter at anthesis, the lobes spreading at anthesis, 8–12 mm long, fleshy or membranous; mature fruits pendulous; pedicels 17–22 mm long; berries conical ovoid or spherical, yellowish green or orange, smooth or slightly rugose	**2**
–	Corollas 3–10 mm in diameter at anthesis, the lobes reflexed at anthesis, 3–5 mm long, membranous; mature fruits erect; pedicels 7–10 mm long; berries conical or ovoid, green, the surface very rugose, rarely smooth	**3**
3	Inflorescences with peduncles and pedicels generally dark purple; calyx with linear or narrowly triangular lobes; corolla lobes membranous, narrowly lanceolate, the apex elongate-acuminate; mature berries ovoid-conical, yellowish green, smooth or rugose; seeds ovoid reniform, brown (Peru [Pasco])	***Solanum stellaticalycinum* M.A.Cueva et al.**
–	Inflorescences with peduncles and pedicels generally whitish green; calyx with triangular or deltate lobes; corolla lobes fleshy, if membranous, always broadly triangular, the apex acute; mature berries globose, orange (ovoid and white when immature), always smooth; seeds flattened reniform, whitish cream or pale tan (Ecuador; Peru [Cusco, Huancavelica, Huánuco, Junín. Loreto, Pasco])	***Solanum incurvum* Ruiz & Pav.**
4	Stems cylindrical; leaves distinctly petiolate (Bolivia; Brazil; Colombia; Ecuador; French Guiana; Guyana; Peru [Amazonas, Cajamarca, Cusco, Huánuco, Junín, Loreto, Madre de Dios, Pasco, San Martín, Ucayali]; Suriname)	***Solanum anceps* Ruiz & Pav.**
–	Stems strongly angular or winged; leaves strongly decurrent onto the stems, sessile (Peru [San Martín])	***Solanum angustialatum* Bitter**
5	Climbing herbs or woody vines	**6**
–	Terrestrial herbs or wand-like subshrubs, occasionally in large colonies	**7**
6	Woody or semi-woody vines, the basal stems to several cm in diameter; flowers 1.6–2 cm in diameter, pinkish tinged or white; petals spreading, fleshy; fruit globose, the surface smooth (Bolivia; Colombia; Ecuador; Peru [Amazonas, Cajamarca, Cusco, Huánuco, Junín, Pasco, San Martín, Ucayali]); Venezuela)	***Solanum ternatum* Ruiz & Pav.**
–	Herbaceous vines, often adhering to tree trunks; flowers 0.6–1 cm in diameter, greenish white; petals strongly reflexed, membranous; fruit conical, the surface rugose (Brazil; Colombia; Ecuador; Peru [Cusco, Huánuco, Junín, Loreto, Madre de Dios, Pasco, San Martín, Ucayali])	***Solanum uleanum* Bitter**
7	Leaves with 5 or fewer pairs of lateral leaflets; leaflets usually obovate and widest in the distal third, especially the terminal leaflet	**8**
–	Leaves with more than 5 pairs of lateral leaflets; lateral leaflets lanceolate to elliptic; terminal leaflet similar in shape to the laterals	**11**
8	Leaves pubescent on veins and lamina on both surfaces	**9**
–	Leaves glabrous on lamina, occasionally pubescent along the veins and rachis	**10**
9	Fruit conical, the surface slightly rugose; leaf pubescence denser adaxially; basal leaflet pair markedly smaller than the other lateral leaflets (Ecuador; Peru [Amazonas, Pasco, San Martín])	***Solanum savanillense* Bitter**
–	Fruit globose or occasionally apically pointed, the surface smooth; leaves equally pubescent on both surfaces; lateral leaflets all more or less the same size (Bolivia; Brazil; Colombia; Ecuador; Peru [Cajamarca, Cusco, Huánuco, Junín, Loreto, Madre de Dios, Pasco, San Martín, Ucayali])	***Solanum mite* Ruiz & Pav.**
10	Fruit conical, the surface rugose; leaves fleshy; plants often rooting at the nodes, very small (Peru [Amazonas, Pasco, San Martín])	***Solanum chamaepolybotryon* Bitter**
–	Fruit globose or occasionally apically pointed, the surface smooth; leaves membranous; plants often semi-woody and up to 1 m tall (Bolivia; Brazil; Colombia; Ecuador; Peru [Cajamarca, Cusco, Huánuco, Junín, Loreto, Madre de Dios, Pasco, San Martín, Ucayali])	***Solanum mite* Ruiz & Pav.**
11	Flowers 0.5–0.6 cm in diameter, the corolla lobes strongly reflexed at anthesis; fruit globose or occasionally apically pointed (Bolivia; Brazil; Colombia; Ecuador; Peru [Cajamarca, Cusco, Huánuco, Junín, Loreto, Madre de Dios, Pasco, San Martín, Ucayali])	***Solanum mite* Ruiz & Pav.**
–	Flowers 0.9–1.3 cm in diameter, the corolla lobes usually spreading or only slightly reflexed at anthesis; fruit conical, rugose	**12**
12	Leaflets with petiolules 3–17 mm long; leaflets somewhat oblique and truncate at the base; leaves densely pubescent in a groove along the rachis adaxially; flowers usually more than 1 cm in diameter (Ecuador; Peru [Amazonas, Cajamarca, Cusco, Huánuco, Madre de Dios, Pasco, San Martín, Ucayali])	***Solanum conicum* Ruiz & Pav.**
–	Leaflets sessile or with short petiolules ca. 1 mm long; leaflets acute to attenuate at the base; leaves glabrous or if on sparsely pubescent then only abaxially; flowers less than 1 cm in diameter (Costa Rica, Guatemala, Mexico, Panama, Venezuela)	***Solanum trizygum* Bitter**

## Supplementary Material

XML Treatment for Solanum stellaticalycinum

## ﻿Additional information

### Use of AI

No use of AI was reported.

### Author ORCIDs

Italo F. Treviño https://orcid.org/0000-0002-2406-7862

Diego A. Sotomayor https://orcid.org/0000-0003-0534-5625

Sandra Knapp https://orcid.org/0000-0001-7698-3945

### Data availability

All of the data that support the findings of this study are available in the main text.
